# Supplementary material for: Identification of photoperiod-induced specific miRNAs in the adrenal glands of Sunite sheep (Ovis aries)
Source: Front Vet Sci. 2022 Jul 22;9:888207. doi: 10.3389/fvets.2022.888207 (PMC9354845; doi:10.3389/fvets.2022.888207)
Supplement: Supplementary file 1 [file Data_Sheet_1.ZIP › Supplementary file/miRDeep2 file.zip!/miRDeep2 file/Know_Structure/LP42A2/oar-mir-106b.pdf]

miRBase precursor : oar-mir-106b
Total read count : 4708
oar-miR-106b read count : 2162
remaining reads : 2546

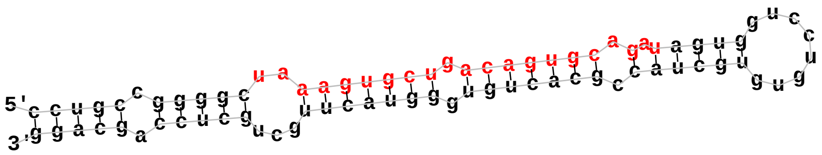

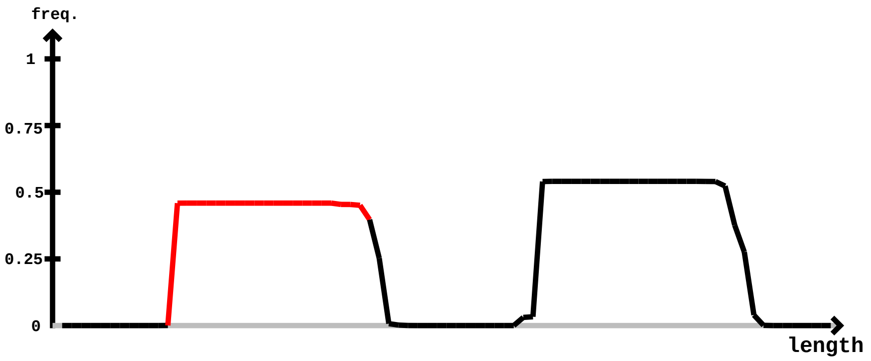


```
             oar-miR-106b
5'- ccugccggggcuaaagugcugacagugcagauagugguccugugugcuaccgcacuguggguacuugcugcuccagcagg  -3'   exp
    (((((.(((((..(((((((.(((((((.(.(((((........)))))).))))))).)))))))...))))).)))))   reads   mm   sample
    ..........cuaaagugcugacagugcagaua...............................................   2       0    seq
    ...........uaaagugcugacagugA....................................................   3       1    seq
    ...........uaaagugcugacaguUc....................................................   1       1    seq
    ...........uaaagugcugacagugc....................................................   19      0    seq
    ...........uaaagugcugacagugca...................................................   1       0    seq
    ...........uaaagugcugacagugcag..................................................   15      0    seq
    ...........uaaagugcugacagugcaga.................................................   251     0    seq
    ...........uaaagugcugacagugcaCa.................................................   1       1    seq
    ...........uaaagugcugacagugcagau................................................   680     0    seq
    ...........uaaagugcugacagugcagaua...............................................   1154    0    seq
    ...........uaaagugcugacagugcagauag..............................................   25      0    seq
    ...........uaaagugcugacagugcagauagu.............................................   8       0    seq
    ...........uaaagugcugacagugcagauagug............................................   1       0    seq
    ............aaagugcugacagugcaga.................................................   1       0    seq
    ...............................................uaccgcacuguggguacuu..............   1       0    seq
    ...............................................uaccgcacuguggguacuugc............   8       0    seq
    ...............................................uaccgcacuguggguacuugcu...........   132     0    seq
    ...............................................uaccgcacuguggguacuugcug..........   1       0    seq
    ...............................................uaccgcacuguggguacuugcugc.........   2       0    seq
    ................................................accgcacuguggguacuugc............   1       0    seq
    ................................................accgcacuguggguacuugcu...........   5       0    seq
    ................................................accgcacuguggguacuugcug..........   1       0    seq
    ................................................accgcacuguggguacuugcugc.........   5       0    seq
    .................................................ccgcacuguggguacuu..............   1       0    seq
    .................................................ccgcacuguggguacuug.............   1       0    seq
    .................................................ccgcacuguggguacuugc............   71      0    seq
    .................................................ccgcacuguggguacuugcu...........   553     0    seq
    .................................................ccgcacuguggguacuugcug..........   469     0    seq
    .................................................ccgcacuguggguacuugcugc.........   1106    0    seq
    .................................................ccgcacuguggguacuugcugcu........   182     0    seq
    .................................................ccgcacuguggguacuugcugcuc.......   4       0    seq
    ..................................................cgcacuguggguacuugcug..........   2       0    seq
    ..................................................cgcacuguggguacuugcugc.........   1       0    seq
```
